# Therapeutic Intensification Based on Immune Checkpoint Inhibitors in Non-Muscle Invasive Bladder Cancer: State of the Art and Future Perspectives

**DOI:** 10.3390/cancers17213555

**Published:** 2025-11-02

**Authors:** Pierre-Etienne Gabriel, Amir Horowitz, Felix Guerrero-Ramos, Francesco Soria, Marco Moschini, David D’Andrea, Benjamin Pradère, John P. Sfakianos, Evanguelos Xylinas

**Affiliations:** 1 Department of Urology, Bichat Claude-Bernard Hospital, Assistance Publique-Hôpitaux de Paris Nord, University Paris Cité, 75018 Paris, France; 2 Department of Oncological Sciences, Tisch Cancer Institute, Icahn School of Medicine at Mount Sinai, New York, NY 10029, USA; 3 Department of Urology, 12 de Octubre University Hospital, Av Córdoba s/n, 28041 Madrid, Spain; felix.guerrero@salud.madrid.org; 4 Department of Urology, Hospital Universitario HM Sanchinarro, 28050 Madrid, Spain; 5 Division of Urology, AOU Citta della Salute e della Scienza di Torino, Torino School of Medicine, 10126 Torino, Italy; 6 Department of Urology, San Raffaele Hospital, Istituto di Ricovero e Cura a Carattere Scientifico (IRCCS), 20132 Milan, Italy; 7 Department of Urology, Medical University of Vienna, 1090 Vienna, Austria; 8 Department of Urology, La Croix Du Sud Hospital, 31130 Quint Fonsegrives, France; 9 Department of Urology, Icahn School of Medicine at Mount Sinai, New York, NY 10029, USA

**Keywords:** non-muscle invasive bladder cancer, high-risk, BCG-naïve, BCG-unresponsive, immunotherapy, PD-1, PD-L1, HLA-E, NKG2A

## Abstract

Background/Objectives: Systemic immunotherapy, previously used mainly for advanced urothelial carcinoma, now plays an important role in the treatment of non-muscle invasive bladder cancer (NMIBC), either alone or combined with intravesical BCG instillations. Methods: We conducted a collaborative, comprehensive review to summarize the key evidence and future perspectives on therapeutic intensification strategies involving immune checkpoint inhibitors in NMIBC. A total of 51 references published between 2000 and 2025 were included. Results: Four phase II studies evaluated pembrolizumab, atezolizumab, durvalumab, and cetrelimab as monotherapy in 28 to 132 BCG-unresponsive NMIBC patients. They reported complete response rates ranging from 12% to 43% after 3 to 12 months of treatment, with a durable response rate ranging from 49% to 57.4% at 12 months. To improve these results, a phase II trial launched this year tests a new systemic combination targeting both the PD-1/PD-L1 axis and the emerging HLA-E/NKG2A pathway. Regarding BCG-naïve high-risk (HR) NMIBC, four phase III studies are evaluating BCG instillations combined with systemic immunotherapy: sasanlimab (CREST), durvalumab (POTOMAC), atezolizumab (ALBAN), and pembrolizumab (KEYNOTE-676), with significant results reported for the CREST and POTOMAC trials. The key challenge remains selecting patients most likely to benefit from this combination therapy while avoiding overtreatment. Identifying predictive biomarkers of tumor aggressiveness and response to immunotherapy also represents a major future challenge. Conclusions: Therapeutic intensification using systemic immunotherapy applies to both BCG-unresponsive NMIBC, with a new target pathway (HLA-E/NKG2A), and BCG-naïve HR NMIBC, where the combination of BCG instillations and immunotherapy represents a major breakthrough.

## Simple Summary

Systemic immunotherapy is now playing an increasingly important therapeutic role in the treatment option of non-muscle invasive bladder cancer (NMIBC), either alone or in combination with BCG instillations. Four phase II studies evaluating PD-1/PD-L1 inhibitors as monotherapy in BCG-unresponsive NMIBC reported complete response rates ranging from 12% to 43%, with durable responses in nearly half of patients at 12 months. A new phase II trial tests a combination therapy targeting both the PD-1/PD-L1 axis and the emerging HLA-E/NKG2A pathway. In BCG-naïve high-risk NMIBC, four phase III trials are evaluating the combination of BCG with systemic immunotherapy, with positive results reported in the CREST and POTOMAC trials, marking a potential therapeutic breakthrough. The main future challenge lies in selecting patients most likely to benefit from this intensified strategy, while avoiding overtreatment, and to find predictive biomarkers for personalized therapy.

## 1. Introduction

Bladder cancer (BC) is the tenth most common cancer worldwide, with an estimated 600,000 new cases annually [1]. At diagnosis, approximately 75% of tumors are non-muscle-invasive, while 25% present with muscle invasion [2]. Non-muscle-invasive bladder cancer (NMIBC) is a heterogeneous disease that can be stratified into low-, intermediate-, high-risk (HR) and very high-risk groups based on the risk of recurrence and progression to muscle-invasive bladder cancer (MIBC) [3]. The standard management of NMIBC consists of transurethral resection of the bladder tumor (TURBT) followed by intravesical chemotherapy or immunotherapy [4]. To date, Bacillus Calmette–Guérin (BCG) immunotherapy remains the gold standard adjuvant treatment for HR-NMIBC, and it is also recommended for intermediate-risk patients [4].

First introduced in 1976 by Alvaro Morales, BCG represents the earliest form of intravesical immunotherapy and has demonstrated high response rates when properly administered, particularly with a six-week induction regimen, ideally followed by a three-year maintenance schedule [5,6]. Despite its efficacy, the BCG treatment regimen has long been challenged—both in terms of the number of instillations [7] and the administered dose [8]—with the aim of reducing side effects and improving treatment tolerability and acceptability. Unfortunately, these trials yielded negative results, leading to the continued use of the conventional BCG treatment regimen.

More recently, molecular studies have identified new targeted options and suggested that involvement of the PD-1/PD-L1 [9] and HLA-E/NKG2A [10,11] pathways could represent major therapeutic targets to restore immune system function and enhance anti-tumor responses. This has prompted the introduction of systemic immunotherapy, either alone for patients with BCG-unresponsive NMIBC or in combination with intravesical BCG installations for BCG-naïve HR-NMIBC patients, as an innovative therapeutic strategy aimed at enhancing treatment efficacy and delaying, or even avoiding, radical cystectomy (RC).

Against this backdrop, we aimed to summarize the key evidence on therapeutic intensification strategies involving immune checkpoint inhibitors in NMIBC.

## 2. Materials and Methods

Starting in May 2025, a comprehensive review using two separate online research platforms (PubMed and Google Scholar) was conducted to retrieve relevant studies on therapeutic intensification based on immune checkpoint inhibitors in NMIBC. We also consulted both the ClinicalTrials.gov database and UroToday to identify and summarize relevant ongoing studies. The literature search employed the following keywords, individually or in combination: “non-muscle invasive bladder cancer”; “high-risk”; “BCG-naïve”; “BCG-unresponsive”; “immunotherapy”; “PD-1”; “PD-L1”; “HLA-E” and “NKG2A”. The search was limited to English-language studies involving human subjects published between 2000 and 2025. After screening, 51 studies met the inclusion criteria and were included in this review. Three authors (P.-E.G., A.H.; E.X.) conducted the selection process to ensure impartial decisions.

A narrative review format was selected to provide a broad synthesis of the existing evidence, considering the heterogeneity of study designs and patient populations in the current literature. This approach enables a comprehensive overview while avoiding the rigid methodological constraints of a systematic review.

## 3. Evidence Synthesis

### 3.1. BCG-Unresponsive NMIBC

Although BCG treatment achieves a complete response in approximately 80% of patients, half of them will experience a recurrence within five years, with progression to MIBC occurring in 20 to 40% of cases [12,13]. Moreover, despite adequate BCG administration, over 40% of patients will become BCG-unresponsive [14]. For these patients, RC remains the only validated therapeutic strategy in Europe [4]. However, this particularly invasive procedure is often difficult to consider for elderly patients or those with comorbidities [15]. Additionally, RC significantly impacts quality of life and body image, which contributes to its limited acceptability [16]. This situation underscores the urgent need to develop alternative bladder-preserving strategies.

From a molecular perspective, preliminary data highlighted high expressions of PD-L1 and HLA-E in BCG-unresponsive NMIBC, representing a therapeutic vulnerability that can be clinically targeted. Indeed, BCG-unresponsive tumors are first characterized by increased interferon (IFN)-γ signaling, resulting from repeated BCG exposure and prolonged activation of type 1 helper T cells (Th1), analogous to immune dysregulation and functional exhaustion observed in microbial infections or chronic inflammation [17,18,19,20,21]. This increased IFN-γ signaling in the tumor microenvironment induces overexpression of HLA-E and PD-L1 by cancer cells, NKG2A on CD8 T cells and natural killer (NK) cells, as well as PD-1 on CD8 T cells [10,22]. Thus, these data support a model of tumor recurrence or progression despite BCG therapy, highlighting the PD-1/PD-L1 and HLA-E/NKG2A axes as immune checkpoints that may be exploited in this context.

Regarding the PD-1/PD-L1 axis, four phase II studies evaluated systemic immunotherapy as monotherapy (pembrolizumab, atezolizumab, durvalumab, and cetrelimab) in 28 to 132 BCG-unresponsive patients [23,24,25,26,27]. Complete response rates ranged from 12% to 43% after 3 to 12 months of treatment, with major adverse events occurring in 7% to 41% of patients (Table 1). KEYNOTE-057 is the first trial to evaluate immunotherapy in BCG-unresponsive NMIBC. This single-arm, multicenter phase II study assessed pembrolizumab (a monoclonal IgG4 anti-PD-1 antibody) administered intravenously at a dose of 200 mg every three weeks for 24 months in 101 patients with BCG-unresponsive CIS (Cohort A) and 132 patients with BCG-unresponsive papillary tumors without CIS. In the CIS cohort, a complete response was achieved in 41% of patients at 3 months, with ongoing response observed in 57.4% at 12 months and 41.3% at 18 months. Disease progression was reported in 9.4% of patients. Regarding safety, 14% of patients experienced grade ≥ 3 adverse events, while 13% had to stop treatment because of side effects; however, no treatment-related deaths were reported. Based on this evidence, systemic pembrolizumab was granted FDA approval in 2020 for patients with BCG-unresponsive CIS, with or without papillary tumors, who were deemed ineligible for RC, thereby introducing systemic immunotherapy for the first-time in the treatment of NMIBC [27]. In the cohort including papillary BCG-unresponsive NMIBC only, the 12-month disease-free survival (DFS) of high-risk NMIBC or progressive disease was 43.5%, with a median DFS of 7·7 months. Treatment-related adverse events occurred in 73% including 14% with grade ≥ 3 [23]. A second multicenter phase II study (SWOG S1605) also reported positive results with systemic atezolizumab, a humanized IgG1 monoclonal antibody that targets PD-L1, administered every three weeks for 12 months in 129 BCG-unresponsive NMIBC (including 74 CIS ± Ta/T1 tumor and 55 papillary tumors without CIS). The trial was designed with two co-primary endpoints: the pathological complete response rate at 6 months in patients with CIS (defined as the absence of high-grade urothelial carcinoma on bladder biopsy and the absence of evidence of upper tract or urethral recurrence) and the event-free survival (EFS) at 18 months (defined as the first occurrence of high-grade bladder cancer, high-grade upper tract urothelial carcinoma, high-grade urothelial carcinoma of the prostatic urethra; muscle-invasive disease; clinical evidence of metastatic disease; or death due to any cause). In the cohort with CIS, complete response rates were 43% at 3 months and 27% at 6 months, respectively, with 56% and 45% of these responses maintained at 12 and 18 months.

With regard to the papillary cohort, the 18-month actuarial EFS rate was 49%. A high-grade recurrence was observed in 32 (58.2) patients and three (5.5%) died without recurrence, representing a total of 35 events in 55 patients (64%), with median EFS of 15 months. The safety profile revealed that 14% of patients experienced major adverse events, including 2 deaths [24]. In the same year, Li et al. reported the results of a phase II study evaluating systemic monotherapy with durvalumab, a human IgG1 monoclonal antibody targeting PD-L1, in 17 patients with BCG-unresponsive CIS. The primary endpoint was the complete response rate at 6 months. The study yielded disappointing results, with only 12% (n = 2) of patients achieving a complete response at 6 months with duration of response of 10 and 18 months, respectively. Additionally, nearly 40% of patients experienced major adverse events, highlighting the potential toxicity of systemic immunotherapy [25]. Finally, more recently, the SunRise-1 study was designed as an open-label, randomized phase II clinical trial enrolling 200 patients with BCG-unresponsive CIS, divided into three treatment arms. Cohort 3 evaluated systemic monotherapy with intravenous cetrelimab, a monoclonal antibody, humanized, that targets PD-1, in 28 patients. The complete response rate based on cystoscopy and systematic biopsy was 46.4% per central assessment and 50.0% per investigator assessment. After a median follow-up in responders of 29.2 months, the median duration of response was 8.6 months, with a 12-month rate of 38.5%. In this cohort, the safety profile showed that half (53.6%) of the patients experienced at least one adverse event, including 7.1% with major adverse events [26]. These results were significantly worse than those achieved in the cohort with intravesical TAR-200 alone, that achieved a complete response rate of 82.4%.

Given the limited efficacy of monotherapies targeting the PD-1/PD-L1 axis, recent molecular insights have highlighted the therapeutic potential of simultaneously inhibiting both PD-L1 and NKG2A pathways. Notably, the NKG2A/HLA-E interaction has emerged as a critical immune checkpoint that suppresses the cytotoxic activity of CD8^+^ T cells and NK cells [10,11]. Its ability to modulate immune activity within the tumor microenvironment offers a solid rationale for using combination blockade strategies to boost immune-driven tumor suppression. The first clinical data were reported in lung cancer, where a phase 2 study evaluating combined immunotherapy targeting both the PD-1/PD-L1 and NKG2A/HLA-E pathways demonstrated improved objective response rates in patients with unresectable stage III non-small-cell lung cancer [28], thereby supporting its exploration in bladder cancer. Thus, ENHANCE (Elevated NKG2A and HLA-E Amplify NK/CD8 Checkpoint Engagers) is the first multicenter phase 2 trial evaluating the combination of durvalumab and monalizumab in patients with BCG-unresponsive NMIBC. Monalizumab is a humanized IgG4 monoclonal antibody that specifically targets NKG2A by binding to it and blocking its interaction with HLA-E, as demonstrated both in vivo and in vitro. Durvalumab is a humanized IgG1 monoclonal antibody that targets PD-L1, inhibiting its interaction with PD-1 and thereby disrupting immune checkpoint signaling. Additionally, durvalumab may trigger antibody-dependent cellular cytotoxicity, primarily mediated by NK cells and potentially by monocytes and neutrophils. Eligible patients will be enrolled to receive up to 12 cycles of monthly combination of intravenous monalizumab and durvalumab. The study was initiated in early 2025 and aims to include two cohorts: 43 patients with BCG-unresponsive CIS with or without papillary disease (Cohort A), and 17 patients with BCG-unresponsive high-grade papillary carcinoma only (Cohort B).

The primary endpoint of the study focuses on Cohort A and is the 6-month complete response rate, defined as the absence of urothelial carcinoma on systematic bladder biopsies. The purpose of Cohort B is to generate pilot data regarding clinical endpoints and biomarkers related to durvalumab plus monalizumab in patients with high grade papillary NMIBC to inform the design of a larger study.

Finally, two studies have evaluated the combination of innovative intravesical therapy with systemic immunotherapy in BCG-unresponsive NMIBC showing promising results and highlighting the potential of combining systemic and local treatments. CORE-001 is a phase II, single-arm study assessing the potential synergistic efficacy of intravesical cretostimogene grenadenorepvec (CG0070) combined with systemic pembrolizumab in patients with BCG-unresponsive NMIBC with CIS. The treatment protocol consisted of an induction phase with six weekly intravesical instillations of CG0070, followed by three weekly maintenance instillations at 3, 6, 9, 12, and 18 months. At 24 months, the complete response (CR) rate in the intention-to-treat (ITT) population was 54% (95% CI 37–71%). Among patients who achieved CR at 12 months, 95% (19/20) maintained their response for an additional 12 months. Progression-free survival (PFS) at 24 months was 100% (no progression to muscle-invasive or metastatic disease), and cystectomy-free survival (CFS) in the CR subgroup was also 100%. This trial is particularly relevant in the NMIBC context, demonstrating that adding a checkpoint inhibitor to novel intravesical immunotherapy can induce substantial and durable responses in BCG-unresponsive NMIBC [29]. The ADAPT-BLADDER Cohort 4 trial, presented at the 2025 ASCO GU Symposium, evaluated the combination of durvalumab with intravesical gemcitabine and docetaxel (Gem/Doc) in patients with BCG-unresponsive NMIBC. This phase I/II, multicenter study included 40 patients (median age 69 years, 83% male), of whom 48% had high-grade papillary disease (Ta/T1) and 53% had CIS (±papillary). Patients received durvalumab intravenously every four weeks for up to six cycles, along with weekly intravesical Gem/Doc for six weeks, followed by optional monthly maintenance. The combination was well tolerated, with no dose-limiting toxicities and few grade 3 adverse events (5%). No grade 4 treatment-related events were observed. Among 37 evaluable patients, 89% achieved a complete response, with similar rates in CIS (90%) and papillary disease (88%). At the time of analysis, 70% of complete responses were ongoing. Ten NMIBC recurrences (27%) and three MIBC recurrences (8%) were reported [30].

### 3.2. BCG-Naive NMIBC

Given the limited therapeutic options currently available for patients with BCG-unresponsive NMIBC, new approaches combining intravesical BCG instillation with upfront systemic immunotherapy have also been considered. Indeed, recent molecular data suggest that the immunostimulatory effects of BCG could be enhanced by the addition of a second immunologic signal, such as an immune checkpoint inhibitor [31,32]. Based on these findings, early-phase clinical trials have assessed combinations of BCG with durvalumab or atezolizumab, reporting robust antitumor immune responses, inhibition of tumor growth and prolonged patient survival [33,34]. The results observed with these combinations surpass the expected additive effects of the individual treatments, indicating potential synergistic interactions and highlighting interest in this strategy for BCG-naïve HR NMIBC. To date, four phase III trials evaluate this therapeutic combination: CREST (sasanlimab; NCT04165317), POTOMAC (durvalumab; NCT03528694), ALBAN (atezolizumab; NCT03799835) and KEYNOTE-676 (pembrolizumab; NCT03711032); Table 2.

CREST is the first study to report positive results with the combination of intravesical BCG instillations and systemic immunotherapy in patients with BCG-naïve HR NMIBC. This phase III, randomized, multicenter study evaluating the efficacy and safety of the subcutaneous sasanlimab in combination with BCG. A total of 1055 patients with HR-NMIBC were equally randomly assigned to sasanlimab (every 28 days for 2 years) plus BCG (induction plus maintenance for 24 months) or sasanlimab plus BCG (induction period without maintenance) or BCG alone (induction and maintenance for 24 months). Randomization was stratified based on CIS status and geographic region. The primary outcome was a composite criterion (event-free survival; EFS) including recurrence of HR disease, progressive disease, persistence of CIS and death from any cause. The results recently published represent a significant advance in the therapeutic option for BCG-naïve HR NMIBC. In fact, the combination therapy resulted in a 32% decrease in the risk of events compared to BCG alone. This difference was primarily driven by a lower rate of HG recurrence between the two groups (7.4% vs. 15.1%). Regarding the predefined subgroup analysis, younger patients (≤65 years) and those with aggressive disease (pT1 and/or CIS) appeared to derive greater benefit from the combination therapy. Conversely, EFS was similar between the group treated with sasanlimab combined with BCG induction only and the control group, emphasizing the significance of a long-term BCG regimen and the potential synergistic effect between the two treatments. Regarding the safety analysis, between 13.8% and 15.7% of patients treated with sasanlimab experienced grade ≥ 3 immune-related adverse events, highlighting a significant toxicity profile and the importance of precise patient selection to avoid overtreatment [35].

POTOMAC is the second study to report positive results with this combination therapy. This multicenter study assessed the efficacy and safety of intravenous durvalumab in combination with BCG compared with BCG alone. In the final analysis, 1018 patients were randomly allocated to three study arms: durvalumab (1500 mg intravenously once a month for 13 cycles) combined with BCG instillations (induction plus 2 years of maintenance), durvalumab with BCG (induction only) or BCG alone (induction and 2 years of maintenance). The primary endpoint was disease-free survival (DFS), defined as the time from randomization to tumor recurrence (high- or low-grade local recurrence or any metastatic event) or death. The results of the POTOMAC study were recently presented at ESMO 2025. After a median follow-up of 60.7 months, the durvalumab + BCG (induction + maintenance) arm showed a 32% reduction in the risk of recurrence or death compared with BCG alone (HR 0.68; *p* = 0.015), with a 24-month DFS of 86.5% versus 81.6% for BCG alone. No negative impact on overall survival or patient-reported quality of life was observed. Durvalumab + BCG induction-only arm did not demonstrate a significant benefit compared with BCG alone (HR 1.14). Grade 3/4 treatment-related adverse events were reported in 21% of patients receiving durvalumab + BCG (induction + maintenance), 15% for durvalumab + BCG induction-only, and 4% for BCG alone, with no treatment-related deaths [36].

ALBAN is a multicenter phase III study assessing the efficacy and safety of intravenous atezolizumab in combination with BCG compared to BCG alone in patients with BCG-naïve HR-NMIBC. A total of 517 patients were randomly assigned into two cohorts: atezolizumab (1200 mg intravenously every three weeks for 1 year) combined with BCG (induction plus 1-year of maintenance) or BCG alone (1 year). The primary endpoint was recurrence-free survival (RFS) defined as reappearance of disease (high- or low-grade local recurrence or occurrence of any metastasis). The results were recently presented at ESMO 2025. After a median follow-up of 35.3 months, there was no statistically significant difference in event-free survival between the two arms (HR 0.98; *p* = 0.91), and high-grade recurrence-free survival was similar (HR 1.06; *p* = 0.77). Overall survival was immature at the data cutoff, with only 25 events reported (HR 1.73). Grade ≥ 3 treatment-related adverse events occurred in 22.7% of patients in the atezolizumab + BCG arm versus 8.8% in the BCG-only arm. The most common adverse events were urinary tract disorders and pollakiuria in the combination arm, and cystitis in the BCG-only arm [37].

To date, KEYNOTE-676 remains the only study assessing the combination of BCG with systemic immunotherapy targeting the PD-1 pathway, and results are not yet available. This fourth multicenter phase III trial compares the efficacy and safety of intravenous pembrolizumab plus BCG versus BCG alone in HR NMIBC.

Approximately 1000 patients were randomly assigned in equal numbers to three cohorts to receive either intravenous pembrolizumab every 3 weeks (for approximately 2 years) combined with BCG therapy (induction plus 3-years of maintenance) or pembrolizumab plus BCG reduced maintenance (induction followed by a single maintenance cycle) or BCG monotherapy alone (3 years). The primary end point was EFS defined as the time from randomization to the first occurrence of high-grade Ta or CIS, any T1 disease of the bladder, high-risk disease (high-grade Ta, CIS, or ≥T1) of the urethra or upper tract, or progression to locally advanced or metastatic disease or death from any cause. The results of this trial have yet to be reported, with publication expected shortly. This publication will be crucial, as it could either reinforce the consistency of data supporting systemic immunotherapy (CREST and POTOMAC) in this indication or cast doubt on its true therapeutic relevance (ALBAN).

Finally, a fifth study (SUNRISE 3; NCT05714202) was recently designed to evaluate the efficacy of systemic anti-PD-1 immunotherapy, alone or in combination with intravesical chemotherapy (gemcitabine) delivered via the new TAR-200 device, in patients with BCG-naïve HR NMIBC. Approximately 1050 patients were randomly assigned to three arms: TAR-200 alone; TAR-200 plus cetrelimab or standard therapy (BCG for 3 years). The primary endpoint is EFS. This study is the first to evaluate the combination of systemic immunotherapy with intravesical chemotherapy, without including BCG in the treatment intensification arm. It could represent an interesting therapeutic alternative to the current standard of care.

## 4. Discussion

Given that systemic immunotherapy is becoming an increasingly important treatment option for NMIBC, we aimed to provide a comprehensive review of the key evidence and future perspectives on therapeutic intensification strategies involving immune checkpoint inhibitors in both BCG-unresponsive and BCG-naïve high-risk NMIBC.

With regard to BCG-unresponsive patients, current data on systemic immunotherapy alone is limited, mainly focusing on tumors with CIS, and the results so far have been modest. Only a few small studies exist, most using complete response rate as the primary endpoint, a different criterion compared to endpoints like event-free survival or disease-free survival used in BCG-naïve trials, especially for papillary-only disease [23,24,25,26,27]. Overall, complete response rates are low (under 25% at 12 months), and durable responses occur in only about half of cases. In addition, systemic immunotherapy can cause significant, sometimes life-conditioning long-lasting side effects, with unclear benefits for many patients. To improve outcomes, ongoing trials are evaluating combinations of two immunotherapy drugs targeting different immune pathways (PD-1/PD-L1 and HLA-E/NKG2A axis).

With regard to BCG-naïve HR NMIBC, two large phase III trials—CREST and POTOMAC—have shown encouraging results. These trials evaluated the combination of anti–PD-1/PD-L1 immunotherapy with intravesical BCG instillations. The CREST trial, the first to be published, represents a key milestone: it is the first positive study to demonstrate the benefit of treatment intensification using checkpoint inhibitors in this setting [35]. The combination of BCG with subcutaneous sasanlimab reduced the high-grade recurrence rate by half. However, this benefit was associated with notable toxicity. In fact, the addition of sasanlimab was associated with 29.1% major treatment related adverse events (grade ≥ 3), including 15.7% of severe immune-mediated adverse events (grade ≥ 3), compared to 6.3% with BCG induction + maintenance alone. The POTOMAC and ALBAN trials both reported a similar signal of increased toxicity associated with the addition of systemic immunotherapy [36,37]. The systemic toxicity adds to the typical side effects already associated with BCG instillations and introduces additional logistical challenges, albeit not as relevant as those posed by intravenous therapies. Still, these concerns need to be weighed against current treatment guidelines. In case of recurrence, RC is often recommended: a major surgery with serious functional consequences. This raises a key question: which patients truly benefit from treatment intensification, and who could be overtreated? In fact, BCG alone remains an effective therapy for many patients HR NMIBC. When administered correctly, 1- and 2-year recurrence-free survival rates reach 89% and 85%, respectively [7], which challenges the need for systematic intensification. Subgroup analysis of the CREST trial suggests that patients with very high-risk features, particularly those with CIS, are most likely to benefit from the combination approach, with durable responses exceeding 90% at 3 years. On the other hand, the management of high-grade Ta tumors remains a major challenge. These tumors accounted for only 27–34% of cases in CREST/POTOMAC study, whereas they represent the majority of HR NMIBC diagnoses [38]. Additionally, over 94% of patients in CREST trial had pure urothelial carcinomas, providing little insight into the effectiveness of this intensified approach in patients with histological subtypes (e.g., micropapillary, plasmacytoid, or sarcomatoid). Future trial designs will be crucial in addressing these unanswered questions.

In both BCG-unresponsive and BCG-naïve NMIBC, the use of systemic immunotherapy to intensify treatment raises two main issues. First, we need clear treatment goals for each risk group. These should consider both the complete response rate, as well as the duration of the response, and the rate of serious side effects, helping to define a cutoff where benefits outweigh harms. Kate et al. reported at the American Society Of Clinical Oncology congress 2023 that the cutoff is a moving target and depends on both the disease state and the existing comparator. They reported a minimal cutoff of 16 in BCG-naive HR NMIBC and 3.6 in BCG-unresponsive NMIBC [39]. Second, we need better tools to select patients most likely to benefit and respond to immunotherapy. This involves improving clinical and pathological criteria (including a more precise classification of the Ta stage) and identifying biomarkers in tissue, blood, or urine that can predict tumor aggressiveness or treatment response. In fact, despite immune checkpoint inhibitors have shown clear clinical benefits in urothelial carcinoma [40,41,42,43,44,45], we still lack a reliable predictive biomarker. PD-L1 expression is the most studied, with various assays and scoring systems employed in clinical trials. Higher PD-L1 levels are generally associated with better responses to systemic immunotherapy, but some PD-L1–negative tumors also respond, limiting its utility as a binary predictive marker [46]. Tumor mutational burden (TMB) is another potential biomarker, as higher TMB may increase neoantigen production and tumor immunogenicity, leading to a stronger immune response [47]. Molecular subtypes of bladder cancer, like luminal and basal/squamous, defined through comprehensive genomic analysis, are also being explored for their predictive value with immunotherapy response [48]. Additionally, a recent study reported the HLA-B-21M/T was independently associated with bladder patient outcomes and can help to optimize the use of new immunotherapies in these patients [49]. Other promising biomarkers include non-invasive urine-based biomarker panel for predicting bladder cancer immunotherapy response [50], tumor-infiltrating lymphocytes, gene expression signatures of inflammation or immune activation, and gut microbiome composition [51]. Further progress will depend on better understanding how the immune system interacts with tumors microenvironment and identifying new therapeutic targets, like the HLA-E/NKG2A pathway, which is drawing increasing attention for its role in response to BCG and other immunotherapies.

## 5. Conclusions

Systemic checkpoint inhibitors appear to be a promising approach, though likely insufficient on their own for the management of BCG-unresponsive NMIBC. In contrast, the combination with intravesical BCG instillations shows encouraging results in BCG-naïve HR NMIBC, as highlighted by recent phase III trial results. However, a key challenge remains in identifying which patients are most likely to benefit from this intensified systemic approach to avoid overtreatment. Developing predictive biomarkers, whether tissue-, blood-, or urine-based—represents a critical area for future research to better personalize treatment strategies.

## Figures and Tables

**Table 1:** Studies evaluating systemic immunotherapy in BCG-unresponsive non-muscle invasive bladder cancer.

	KEYNOTE 057 FDA Approved	SWOG S1605	Phase-2 Durvalumab	SunRise-1 Cohort 3	ENHANCE
**Immunotherapy**	Pembrolizumab	Atezolizumab	Durvalumab	Cetrelimab	Durvalumab + Monalizumab
**Mechanism**	Anti PD-1	Anti PD-L1	Anti PD-L1	Anti PD-1	Anti PDL-L1 + Anti NKG2A
**Duration of Immunotherapy**	2 years	1 year	1 year	18 months	1 years
**Immunotherapy administration**	Intravenous	Intravenous	Intravenous	Intravenous	Intravenous
**Immunotherapy schedule**	Every 3 weeks	Every 3 weeks	Every 4 weeks	Every 3 weeks	Every 4 weeks
**Number of patients**	101 (CIS ± Ta/T1)	129 (74 CIS)	17 (CIS)	28 (CIS)	60 (43 CIS ± Ta/T1 and 17 Ta/T1)
**3 months CR in CIS (%)**	41	43	12 *	/	NA
**12 months CR in CIS (%)**	19	25	/	22.8	NA
**Persistence of CR at 12 months (%)**	57.4	56	50	48.5	NA
**Any all-grade treatment-related AEs, n (%)**	67 (65.7)	81 (97)	17 (100)	15 (53.6)	NA
**Grade 3–5 treatment-related AEs, n (%)**	13 (13)	13 (14)	7 (41)	7.1%	NA
**Discontinuation because of treatment-related AEs, n (%)**	9 (8.8)	9 (9)	0 (0)	1 (4.2)	NA
**Death because of treatment-related AEs, n (%)**	0 (0)	2 (2)	0 (0)	0 (0)	NA

**CR**: complete response; **CIS**: carcinoma in situ; **AEs**: adverse events, **NA**: not available. * 6 months CR.

**Table 2:** Studies evaluating systemic immunotherapy in BCG-naïve high-risk non-muscle invasive bladder cancer.

	ALBAN	CREST	POTOMAC	KEYNOTE 676	SunRise-3
**Immunotherapy**	Atezolizumab	Sasanlimab	Durvalumab	Pembrolizumab	Cetrelimab
**Mechanism**	Anti PD-L1	Anti PD-1	Anti PD-L1	Anti PD-1	Anti PD-1
**Duration of Immunotherapy**	1 year	2 years	1 year	1 year	2 years
**Administration of Immunotherapy**	Intravenous	Subcutaneous	Intravenous	Intravenous	Intravenous
**Immunotherapy schedule**	Every 3 weeks	Every 4 weeks	Every 4 weeks	Every 3 weeks	Every 3 weeks
**Estimated number of patients**	517	1055	1018	1000	1050
**Treatment arms**	Atezolizumab + BCG (IND + MAIN) vs. BCG control	Sasanlimab + BCG (IND + MAIN) or + BCG (IND) vs. BCG control	Durvalumab + BCG (IND + MAIN) or + BCG (IND) vs. BCG control	Pembrolizumab + BCG (IND + MAIN) vs. Pembrolizumab + BCG (IND + reduced MAIN) vs. BCG control	Cetrelimab + TAR 200 vs. TAR 200 vs. BCG control
**Primary endpoint**	RFS	EFS	DFS	EFS	EFS
**Results for the primary endpoint**	Negative	Positive	Positive	NA	NA

**PD-L1** = programmed cell death-(ligand) 1; **BCG** = bacillus Calmette-Guérin; **IND** = induction; **MAIN** = maintenance; **RFS** = recurrence-free survival; **EFS** = event-free survival; **DFS** = disease-free survival; **CIS** = carcinoma in situ; **NA**: not available. **Definition of endpoints: RFS**: High-grade or low-grade local recurrence or occurrence of any metastasis. **EFS**: High-grade local recurrence, progression of disease, persistence of CIS, or death. **DFS**: High-grade or low-grade local recurrence or occurrence of any metastasis or death.

## Data Availability

No new data were created or analyzed in this study. Data sharing is not applicable to this article.

## References

[1] Richters A., Aben K.K.H., Kiemeney L.A.L.M. (2020). The global burden of urinary bladder cancer: An update. World J. Urol..

[2] Antoni S., Ferlay J., Soerjomataram I., Znaor A., Jemal A., Bray F. (2017). Bladder Cancer Incidence and Mortality: A Global Overview and Recent Trends. Eur. Urol..

[3] Jobczyk M., Stawiski K., Fendler W., Różański W. (2020). Validation of EORTC, CUETO, and EAU risk stratification in prediction of recurrence, progression, and death of patients with initially non-muscle-invasive bladder cancer (NMIBC): A cohort analysis. Cancer Med..

[4] Gontero P., Birtle A., Capoun O., Compérat E., Dominguez-Escrig J.L., Liedberg F., Mariappan P., Masson-Lecomte A., Mostafid H.A., Pradere B. (2024). European Association of Urology Guidelines on Non-muscle-invasive Bladder Cancer (TaT1 and Carcinoma In Situ)-A Summary of the 2024 Guidelines Update. Eur. Urol..

[5] Lamm D.L., Blumenstein B.A., Crissman J.D., Montie J.E., Gottesman J.E., Lowe B.A., Sarosdy M.F., Bohl R.D., Grossman H.B., Beck T.M. (2000). Maintenance bacillus Calmette-Guerin immunotherapy for recurrent TA, T1 and carcinoma in situ transitional cell carcinoma of the bladder: A randomized Southwest Oncology Group Study. J. Urol..

[6] Sylvester R.J., van der Meijden A.P., Lamm D.L. (2002). Intravesical bacillus Calmette-Guerin reduces the risk of progression in patients with superficial bladder cancer: A meta-analysis of the published results of randomized clinical trials. J. Urol..

[7] Grimm M.-O., van der Heijden A.G., Colombel M., Muilwijk T., Martínez-Piñeiro L., Babjuk M.M., Türkeri L.N., Palou J., Patel A., Bjartell A.S. (2020). Treatment of High-grade Non-muscle-invasive Bladder Carcinoma by Standard Number and Dose of BCG Instillations Versus Reduced Number and Standard Dose of BCG Instillations: Results of the European Association of Urology Research Foundation Randomised Phase III Clinical Trial “NIMBUS”. Eur. Urol..

[8] Oddens J., Brausi M., Sylvester R., Bono A., van de Beek C., van Andel G., Gontero P., Hoeltl W., Turkeri L., Marreaud S. (2013). Final results of an EORTC-GU cancers group randomized study of maintenance bacillus Calmette-Guérin in intermediate- and high-risk Ta, T1 papillary carcinoma of the urinary bladder: One-third dose versus full dose and 1 year versus 3 years of maintenance. Eur. Urol..

[9] Fukumoto K., Kikuchi E., Mikami S., Hayakawa N., Matsumoto K., Niwa N., Oya M. (2018). Clinical Role of Programmed Cell Death-1 Expression in Patients with Non-muscle-invasive Bladder Cancer Recurring After Initial Bacillus Calmette-Guérin Therapy. Ann. Surg. Oncol..

[10] Salomé B., Sfakianos J.P., Ranti D., Daza J., Bieber C., Charap A., Hammer C., Banchereau R., Farkas A.M., Ruan D.F. (2022). NKG2A and HLA-E define an alternative immune checkpoint axis in bladder cancer. Cancer Cell.

[11] Ranti D., Yu H., Salomé B., Bang S., Duquesne I., Wang Y.A., Bieber C., Strandgaard T., Merritt E., Doherty G. (2025). HLA-E and NKG2A Mediate Resistance to M. bovis BCG Immunotherapy in Non-Muscle-Invasive Bladder Cancer. BioRxiv.

[12] Van den Bosch S., Alfred Witjes J. (2011). Long-term cancer-specific survival in patients with high-risk, non-muscle-invasive bladder cancer and tumour progression: A systematic review. Eur. Urol..

[13] Sylvester R.J., van der Meijden A.P., Oosterlinck W., Witjes J.A., Bouffioux C., Denis L., Newling D.W., Kurth K. (2006). Predicting recurrence and progression in individual patients with stage Ta T1 bladder cancer using EORTC risk tables: A combined analysis of 2596 patients from seven EORTC trials. Eur. Urol..

[14] Zlotta A.R., Fleshner N.E., Jewett M.A. (2009). The management of BCG failure in non-muscle-invasive bladder cancer: An update. Can. Urol. Assoc. J..

[15] Prayer Galetti T., Soligo M., Morlacco A., Lami V., Nguyen A.A.L., Iafrate M., Zattoni F. (2021). Morbidity, mortality, and quality assessment following open radical cystectomy in elderly patients with bladder cancer. Aging Clin. Exp. Res..

[16] Bahlburg H., Reicherz A., Reike M., Bach P., Butea-Bocu M.C., Tully K.H., Roghmann F., Noldus J., Müller G. (2025). A prospective evaluation of quality of life, psychosocial distress, and functional outcomes two years after radical cystectomy and urinary diversion in 842 German bladder cancer patients. J. Cancer Surviv..

[17] Chowell D., Yoo S.-K., Valero C., Pastore A., Krishna C., Lee M., Hoen D., Shi H., Kelly D.W., Patel N. (2022). Improved prediction of immune checkpoint blockade efficacy across multiple cancer types. Nat. Biotechnol..

[18] Chowell D., Krishna C., Pierini F., Makarov V., Rizvi N.A., Kuo F., Morris L.G.T., Riaz N., Lenz T.L., Chan T.A. (2019). Evolutionary divergence of HLA class I genotype impacts efficacy of cancer immunotherapy. Nat. Med..

[19] Chowell D., Morris L.G.T., Grigg C.M., Weber J.K., Samstein R.M., Makarov V., Kuo F., Kendall S.M., Requena D., Riaz N. (2018). Patient HLA class I genotype influences cancer response to checkpoint blockade immunotherapy. Science.

[20] Chhibber A., Huang L., Zhang H., Xu J., Cristescu R., Liu X., Mehrotra D.V., Shen J., Shaw P.M., Hellmann M.D. (2022). Germline HLA landscape does not predict efficacy of pembrolizumab monotherapy across solid tumor types. Immunity.

[21] Havel J.J., Chowell D., Chan T.A. (2019). The evolving landscape of biomarkers for checkpoint inhibitor immunotherapy. Nat. Rev. Cancer.

[22] Rodríguez-Izquierdo M., Del Cañizo C.G., Rubio C., Reina I.A., Hernández Arroyo M., Rodríguez Antolín A., Dueñas Porto M., Guerrero-Ramos F. (2023). Immune Predictors of Response after Bacillus Calmette–Guérin Treatment in Non-Muscle-Invasive Bladder Cancer. Cancers.

[23] Necchi A., Roumiguié M., Kamat A.M., Shore N.D., Boormans J.L., Esen A.A., Lebret T., Kandori S., Bajorin D.F., Krieger L.E.M. (2024). Pembrolizumab monotherapy for high-risk non-muscle-invasive bladder cancer without carcinoma in situ and unresponsive to BCG (KEYNOTE-057): A single-arm, multicentre, phase 2 trial. Lancet Oncol..

[24] Black P.C., Tangen C.M., Singh P., McConkey D.J., Lucia M.S., Lowrance W.T., Koshkin V.S., Stratton K.L., Bivalacqua T.J., Kassouf W. (2023). Phase 2 Trial of Atezolizumab in Bacillus Calmette-Guérin-unresponsive High-risk Non-muscle-invasive Bladder Cancer: SWOG S1605. Eur. Urol..

[25] Li R., Sexton W.J., Dhillon J., Berglund A., Naidu S., Borjas G., Rose K., Kim Y., Wang X., Conejo-Garcia J.R. (2023). A Phase II Study of Durvalumab for Bacillus Calmette-Guerin (BCG) Unresponsive Urothelial Carcinoma In Situ of the Bladder. Clin. Cancer Res..

[26] Daneshmand S., Van der Heijden M.S., Jacob J.M., Guerrero-Ramos F., Bögemann M., Simone G., Pieczonka C.M., Canales Casco N., Zainfeld D., Spiegelhalder P. (2025). TAR-200 for Bacillus Calmette-Guérin–Unresponsive High-Risk Non–Muscle-Invasive Bladder Cancer: Results From the Phase IIb SunRISe-1 Study. J. Clin. Oncol..

[27] Balar A.V., Kamat A.M., Kulkarni G.S., Uchio E.M., Boormans J.L., Roumiguié M., Krieger L.E.M., Singer E.A., Bajorin D.F., Grivas P. (2021). Pembrolizumab monotherapy for the treatment of high-risk non-muscle-invasive bladder cancer unresponsive to BCG (KEYNOTE-057): An open-label, single-arm, multicentre, phase 2 study. Lancet Oncol..

[28] Herbst R.S., Majem M., Barlesi F., Carcereny E., Chu Q., Monnet I., Sanchez-Hernandez A., Dakhil S., Camidge D.R., Winzer L. (2022). COAST: An Open-Label, Phase II, Multidrug Platform Study of Durvalumab Alone or in Combination With Oleclumab or Monalizumab in Patients With Unresectable, Stage III Non-Small-Cell Lung Cancer. J. Clin. Oncol..

[29] Li R., Shah P.H., Stewart T.F., Nam J.K., Bivalacqua T.J., Lamm D.L., Uchio E.M., Geynisman D.M., Jacob J.M., Meeks J.J. (2024). Oncolytic adenoviral therapy plus pembrolizumab in BCG-unresponsive non-muscle-invasive bladder cancer: The phase 2 CORE-001 trial. Nat. Med..

[30] ASCO GU 2025: A Phase 1/2 Trial of Durvalumab plus Intravesical Gemcitabine and Docetaxel in BCG-Unresponsive Non-Muscle Invasive Bladder Cancer Patients (HCRN GU16-243: ADAPT-BLADDER Cohort 4). https://www.urotoday.com/conference-highlights/asco-gu-2025/asco-gu-2025-bladder-cancer/158301-asco-gu-2025-a-phase-1-2-trial-of-durvalumab-plus-intravesical-gemcitabine-and-docetaxel-in-bcg-unresponsive-non-muscle-invasive-bladder-cancer-patients-hcrn-gu16-243-adapt-bladder-cohort-4.html.

[31] Lobo N., Brooks N.A., Zlotta A.R., Cirillo J.D., Boorjian S., Black P.C., Meeks J.J., Bivalacqua T.J., Gontero P., Steinberg G.D. (2021). 100 years of Bacillus Calmette-Guérin immunotherapy: From cattle to COVID-19. Nat. Rev. Urol..

[32] van Puffelen J.H., Keating S.T., Oosterwijk E., van der Heijden A.G., Netea M.G., Joosten L.A.B., Vermeulen S.H. (2020). Trained immunity as a molecular mechanism for BCG immunotherapy in bladder cancer. Nat. Rev. Urol..

[33] Hahn N.M., O’Donnell M.A., Efstathiou J.A., Zahurak M., Rosner G.L., Smith J., Kates M.R., Bivalacqua T.J., Tran P.T., Song D.Y. (2023). A Phase 1 Trial of Durvalumab in Combination with Bacillus Calmette-Guerin (BCG) or External Beam Radiation Therapy in Patients with BCG-unresponsive Non-muscle-Invasive Bladder Cancer: The Hoosier Cancer Research Network GU16-243 ADAPT-BLADDER Study. Eur. Urol..

[34] Inman B.A., Hahn N.M., Stratton K., Kopp R., Sankin A., Skinner E., Pohar K., Gartrell B.A., Pham S., Rishipathak D. (2023). A Phase 1b/2 Study of Atezolizumab with or Without Bacille Calmette-Guérin in Patients with High-risk Non-muscle-invasive Bladder Cancer. Eur. Urol. Oncol..

[35] Shore N.D., Powles T.B., Bedke J., Galsky M.D., Palou Redorta J., Ku J.H., Kretkowski M., Xylinas E., Alekseev B., Ye D. (2025). Sasanlimab plus BCG in BCG-naive, high-risk non-muscle invasive bladder cancer: The randomized phase 3 CREST trial. Nat. Med..

[36] De Santis M., Palou Redorta J., Nishiyama H., Krawczyński M., Seyitkuliev A., Novikov A., Guerrero-Ramos F., Zukov R., Kato M., Kawahara T. (2025). Durvalumab in combination with BCG for BCG-naive, high-risk, non-muscle-invasive bladder cancer (POTOMAC): Final analysis of a randomised, open-label, phase 3 trial. Lancet.

[37] Roupret M., Bertaut A., Pignot G., Neuzillet Y., Houede N., Mathieu R., Corbel L., Besson D., Seisen T., Jaffrelot L. (2025). ALBAN (GETUG-AFU 37): A phase 3, randomized, open-label, international trial of intravenous atezolizumab and intravesical Bacillus Calmette-Guérin (BCG) versus BCG alone in BCG-naive high-risk, non-muscle invasive bladder cancer (NMIBC). Ann. Oncol..

[38] Lebret T., Bonastre J., Fraslin A., Neuzillet Y., Droupy S., Rebillard X., Vordos D., Guy L., Villers A., Schneider M. (2023). Cohort profile: COBLAnCE: A French prospective cohort to study prognostic and predictive factors in bladder cancer and to generate real-world data on treatment patterns, resource use and quality of life. BMJ Open.

[39] ASCO GU 2023 Bladder Cancer. https://www.urotoday.com/conference-highlights/asco-gu-2023/asco-gu-2023-bladder-cancer.html.

[40] Bajorin D.F., Witjes J.A., Gschwend J.E., Schenker M., Valderrama B.P., Tomita Y., Bamias A., Lebret T., Shariat S.F., Park S.H. (2021). Adjuvant Nivolumab versus Placebo in Muscle-Invasive Urothelial Carcinoma. N. Engl. J. Med..

[41] van der Heijden M.S., Sonpavde G., Powles T., Necchi A., Burotto M., Schenker M., Sade J.P., Bamias A., Beuzeboc P., Bedke J. (2023). Nivolumab plus Gemcitabine-Cisplatin in Advanced Urothelial Carcinoma. N. Engl. J. Med..

[42] Powles T., Valderrama B.P., Gupta S., Bedke J., Kikuchi E., Hoffman-Censits J., Iyer G., Vulsteke C., Park S.H., Shin S.J. (2024). Enfortumab Vedotin and Pembrolizumab in Untreated Advanced Urothelial Cancer. N. Engl. J. Med..

[43] Powles T., Park S.H., Caserta C., Valderrama B.P., Gurney H., Ullén A., Loriot Y., Sridhar S.S., Sternberg C.N., Bellmunt J. (2023). Avelumab First-Line Maintenance for Advanced Urothelial Carcinoma: Results From the JAVELIN Bladder 100 Trial After ≥2 Years of Follow-Up. J. Clin. Oncol..

[44] Bellmunt J., de Wit R., Vaughn D.J., Fradet Y., Lee J.-L., Fong L., Vogelzang N.J., Climent M.A., Petrylak D.P., Choueiri T.K. (2017). Pembrolizumab as Second-Line Therapy for Advanced Urothelial Carcinoma. N. Engl. J. Med..

[45] Apolo A.B., Ballman K.V., Sonpavde G., Berg S., Kim W.Y., Parikh R., Teo M.Y., Sweis R.F., Geynisman D.M., Grivas P. (2025). Adjuvant Pembrolizumab versus Observation in Muscle-Invasive Urothelial Carcinoma. N. Engl. J. Med..

[46] Rui X., Gu T.-T., Pan H.-F., Zhang H.-Z. (2019). Evaluation of PD-L1 biomarker for immune checkpoint inhibitor (PD-1/PD-L1 inhibitors) treatments for urothelial carcinoma patients: A meta-analysis. Int. Immunopharmacol..

[47] Samstein R.M., Lee C.-H., Shoushtari A.N., Hellmann M.D., Shen R., Janjigian Y.Y., Barron D.A., Zehir A., Jordan E.J., Omuro A. (2019). Tumor mutational load predicts survival after immunotherapy across multiple cancer types. Nat. Genet..

[48] Robertson A.G., Kim J., Al-Ahmadie H., Bellmunt J., Guo G., Cherniack A.D., Hinoue T., Laird P.W., Hoadley K.A., Akbani R. (2017). Comprehensive Molecular Characterization of Muscle-Invasive Bladder Cancer. Cell.

[49] Ruiz-Lorente I., Gimeno L., López-Abad A., López Cubillana P., Fernández Aparicio T., Asensio Egea L.J., Moreno Avilés J., Doñate Iñiguez G., Guzmán Martínez-Valls P.L., Server G. (2025). Differential Role of NKG2A/HLA-E Interaction in the Outcomes of Bladder Cancer Patients Treated with *M. bovis* BCG or Other Therapies. Biomedicines.

[50] Guerrero-Ramos F., Suárez-Cabrera C., Gómez-Canizo C., Hernández-Arroyo M., Martín-Rodríguez R., Gervás-Yubero C., Martín-Bernardo Á., Castellano D., Jesús P., Rodríguez-Antolín A. (2025). Ip14-31 bladimirplus: A non-invasive urine-based biomarker panel for predicting bladder cancer immunotherapy outcomes. J. Urol..

[51] Kato M., Uchida J. (2023). Recent advances in immune checkpoint inhibitors in the treatment of urothelial carcinoma: A review. Int. J. Urol..

